# Delayed presentation of a huge abdominopelvic mass during the COVID-19 pandemic

**DOI:** 10.1016/j.amsu.2022.104576

**Published:** 2022-09-12

**Authors:** Rajan Gurung, Aishath Azna Ali, Firdaus Hayati, Vishnu Vinodhan Rajakumar, Alvin Oliver Payus, Aye Aye Wynn, Nornazirah Azizan, Mohsen Mohamed Ahmed Abdelhafez, Bahiyah Abdullah

**Affiliations:** a Department of Surgery, Indira Gandhi Memorial Hospital, Male, Maldives; b Department of Surgery, Faculty of Medicine and Health Sciences, Universiti Malaysia Sabah, Kota Kinabalu, Sabah, Malaysia; c Department of Medicine, Faculty of Medicine and Health Sciences, Universiti Malaysia Sabah, Kota Kinabalu, Sabah, Malaysia; d Department of Pathology and Microbiology, Faculty of Medicine and Health Sciences, Universiti Malaysia Sabah, Kota Kinabalu, Sabah, Malaysia; e Department of Obstetrics and Gynaecology, Faculty of Medicine and Health Sciences, Universiti Malaysia Sabah, Kota Kinabalu, Sabah, Malaysia; f Department of Obstetrics and Gynaecology, Faculty of Medicine, Universiti Teknologi MARA, Sungai Buloh Campus, Sungai Buloh, Selangor, Malaysia

**Keywords:** Abdominal neoplasms, COVID-19, Critical care, Mucinous cystadenoma, Pandemic

## Abstract

**Background:**

Giant ovarian cysts are rare in developed countries due to advanced achievements in medical diagnostics. However, in the midst of the coronavirus disease 2019 (COVID-19) pandemic, patients with non-COVID-19-related illnesses tend to delay their health-seeking attention; thus, they had presented late.

**Case presentation:**

A 25-year-old single lady complained of a 3-month worsening abdominal pain and distention. She was initially well but neglected the symptoms due to the COVID-19 situation, yet came to our attention after she developed obstructive symptoms. A computed tomography (CT) scan of the abdomen revealed a huge cystic lesion from the pelvic area, which later was found to be from the right ovary upon urgent laparotomy exploration. The histopathological examination was consistent with mucinous cystadenoma of the ovary.

**Discussion:**

Acute non-COVID-19-related emergencies have decreased, as evidenced by reduced visits to the Emergency Department, and the number of abdominal CT scans. An emergency case like a huge abdominopelvic mass deserves an extensive radiologic examination as clinical assessment alone may not be adequate. Preoperative CT is superior to ultrasonography in getting the extent of the lesion, local infiltration, staging purpose, and surgical intervention. Pathology with a variety of spectrums such as mucinous neoplasm deserves to be investigated, evaluated, and resected even during the COVID-19 pandemic.

**Conclusion:**

A giant abdominopelvic cystic mass can present emergency havoc during the COVID-19 pandemic. Urgent surgical intervention is mandatory by using full protection and exercising extreme precaution, regardless of the preoperative screening to avoid unnecessary viral transmissions.

## Highlights

• Acute abdomen cases have reduced in numbers during COVID-19 pandemic which can cause delay in diagnosis and management.
• Preoperative CT scan can determine the extent of the lesion, local infiltration, staging, and surgical intervention.
• During pandemic, urgent surgical intervention is mandatory by using full protection regardless of the preoperative screening to avoid unnecessary viral transmissions.

## 1 Introduction

Huge abdominal cystic lesions are common cases being referred to emergency surgeries. Occasionally, various diagnoses of cystic lesions can be categorised according to their histologic and anatomical origins [[1], [2], [3]]. Although common, they are rarely discovered in developed countries due to the availability of advanced imaging modalities and enhanced surgical expertise; hence, leading to early diagnoses of smaller-sized tumours. Giant ovarian cysts, which measure more than 10 cm in size, can be neglected due to a variety of reasons [4]. This is inclusive of the current coronavirus disease 2019 (COVID-19) pandemic, which has contributed significantly to diagnostic delays. Giant ovarian cysts rarely manifest in the early stages, subsequently becoming symptomatic due to compression, heaviness, or visible mass, only after reaching a substantial expanse [5]. We report a 25-year-old young lady who presented with compressive symptoms after delaying seeking medical attention due to the COVID-19 pandemic, and we will discuss our management approach. This case report has been reported in line with SCARE 2020 criteria [6].

## 2 Case presentation

A 25-year-old single lady presented with complaints of constipation, recurrent vomiting, and abdominal distension. She initially noticed her abdomen becoming distended three months prior, associated with a mild occasional colicky abdominal pain. She was hesitant to seek medical attention during the initial period due to the evolving COVID-19 pandemic. However, as the symptoms gradually progressed during the last 1 week, in addition to the increasing obstructive features, she reluctantly came to the hospital for consultation.

**Fig. 1:**
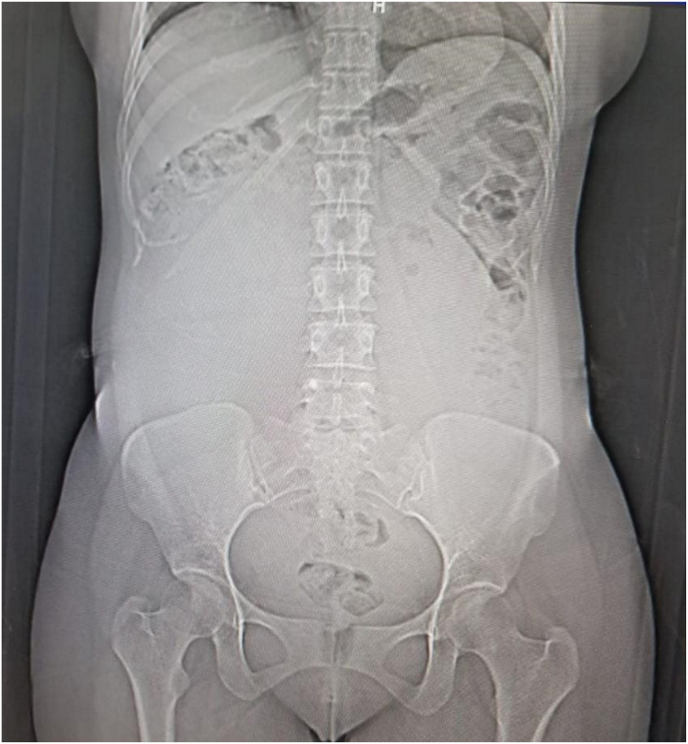
Abdominal radiograph showing a large soft tissue density, which displaced the bowels superiorly, likely to have originated from the pelvis. There is no calcification within the density.

**Fig. 2:**
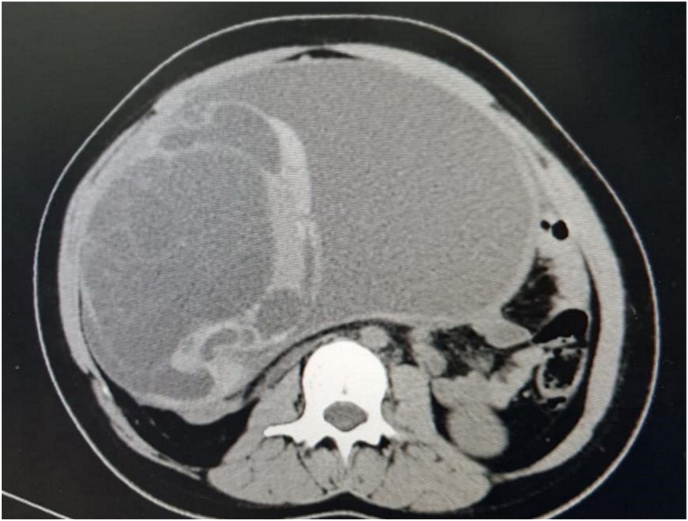
Selected image in the axial section of a contrast CT of the abdomen demonstrating a large complex non-fat containing cystic mass with multiple smaller locules of cysts with enhancing septations within, suggesting a multilocular cystic mass. There is no calcification within.

Upon the initial presentation, she was considered a well-built, nourished lady with no cachexia or dehydration. She was clinically stable. Physical examination revealed a distended, tense abdomen without any sign of peritonism. Blood investigations were within normal range. In view of the possibility of gynaecologic pathology, tumour markers including serum cancer antigen (CA) 125, lactate dehydrogenase (LDH), and carcinoembryonic antigen (CEA), were performed showing normal results. Simple abdominal radiography revealed an extensive radiopaque lesion involving the abdominopelvic region with no evidence of intestinal obstruction (Fig. 1). A computed tomography (CT) scan reported a large 24 x 21 × 12 cm complex cystic lesion from the bladder vault, inferiorly extending up to the transverse colon superiorly. It showed a thin-walled, multiseptated cyst within itself with mural wall thickening (Fig. 2). The surrounding fat planes appeared clean without any evidence of ascites or lymphadenopathy. A diagnosis of peritoneal hydatid cyst was made, after considering the endemic region and the CT features. She was immediately posted for emergency surgery with the diagnosis of a peritoneal hydatid cyst causing obstructive symptoms. Screening for COVID-19 was done prior to surgery by using a throat swab rapid test kit.

**Fig. 3:**
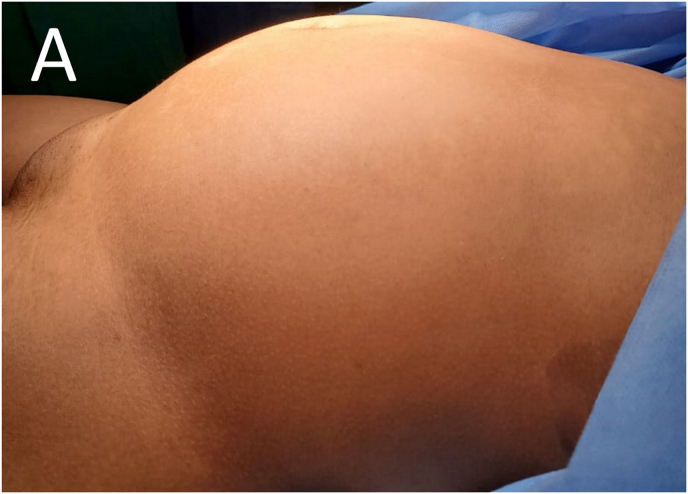

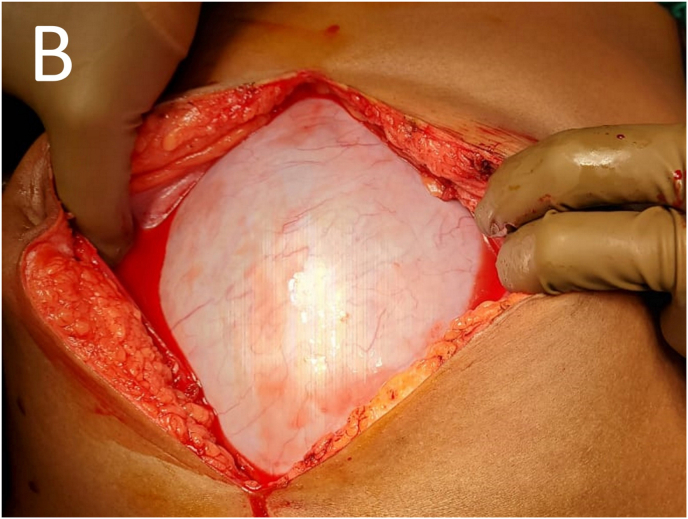

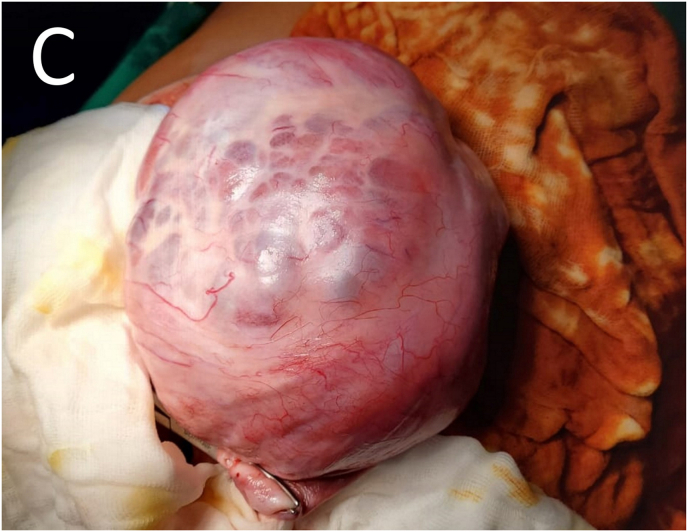

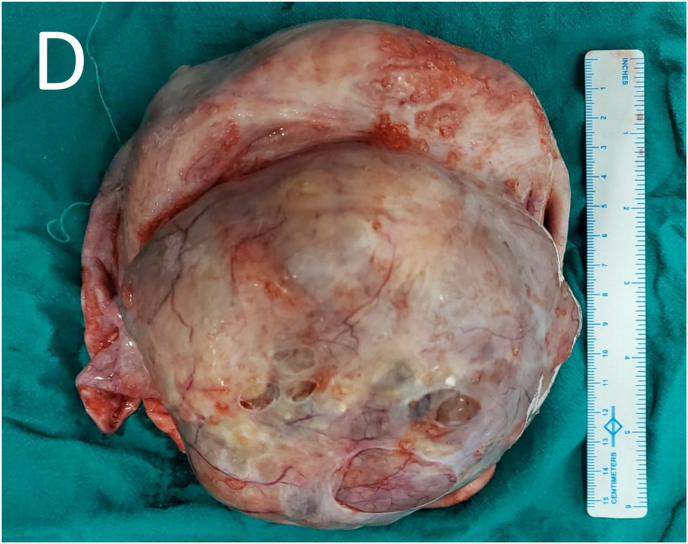
A–D. (A) Grossly distended abdomen when the patient is on the table; (B) Huge mass occupying the entire abdominal cavity, extending from the diaphragm until the pelvis; (C) The mass originating from the right ovary; (D) A greyish white, multiloculated cyst was retrieved.

**Fig. 4:**
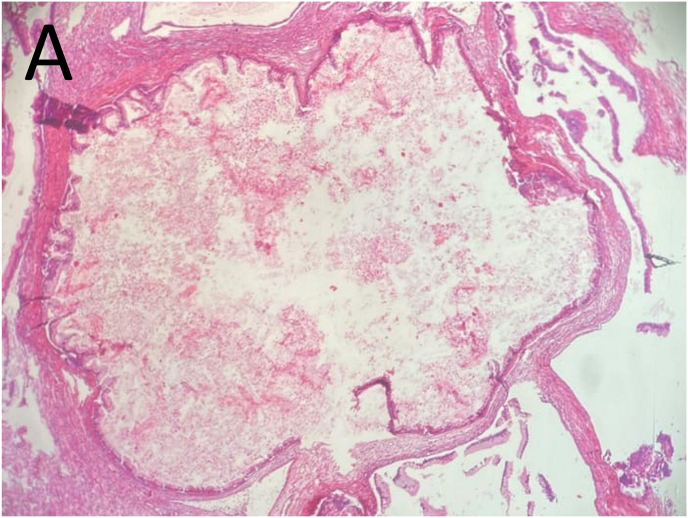

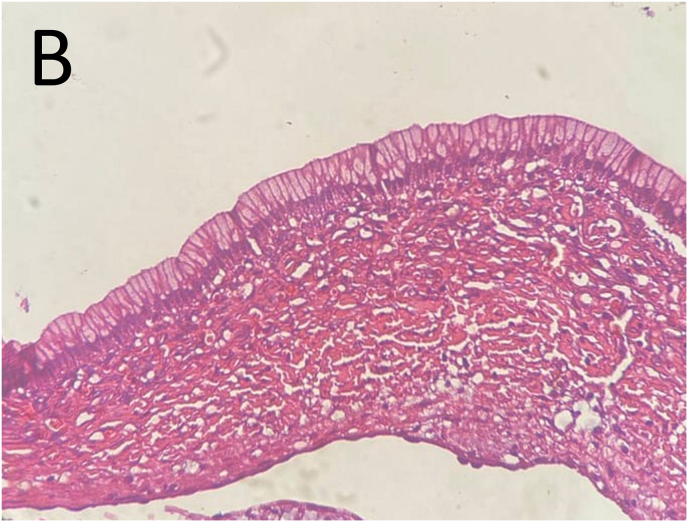
(A) Multiloculated cyst containing mucin, separated by a fibrous septae (haematoxylin and eosin, original magnification x10); (B) Cyst wall lined by a single layer of mucin secreting columnar epithelium (haematoxylin and eosin, original magnification x40).

On laparotomy, a generous midline incision was made extending from the xiphisternum to the pubic symphysis to allow for a better surgical view and access (Fig. 3A). Intraoperatively, there was a huge mass occupying the entire abdominal cavity, extending from the diaphragm until the pelvis (Fig. 3B). In view of the inability to deliver the specimen as a whole, needle aspiration was decided. A total of 2.9 L of clear-coloured fluid content was successfully aspirated. Once collapsed, we were able to trace it as having originated from the right ovary. A gynaecologist was called in intraoperatively and a thorough examination was performed. The rest of the visualised pelvic organs, including the left ovary and the uterus, were normal. Right salpingo-oophorectomy was performed and a specimen was sent for a histopathological examination (Fig. 3C). Specimen of the greyish-white, multiloculated cyst was retrieved macroscopically (Fig. 3D). The histopathological examination was consistent with mucinous cystadenoma of the ovary (Fig. 4). The postoperative period was uneventful, and the patient was discharged well on day 5. Upon follow-up, she was well without any complications.

## 3 Discussion

The COVID-19 pandemic has created global chaos, especially in the healthcare system [7]. Cases of acute non-COVID-19-related emergencies had reduced in number, as evidenced by a significant reduction in the overall number of ED visits, as well as the number of abdominal CT scans performed [[8], [9], [10]]. During the pandemic, non-essential elective surgery cases were deferred, owing to the fear of contracting COVID-19 while in hospital. This panic has caused a delay in seeking medical attention, thus increasing the rate of complications [11]. Ideally, all cases should be done during elective settings and in dedicated COVID-19-free surgical pathways during the COVID-19 pandemic [12]. Postoperative SARS-CoV-2 infection and pulmonary complication rates were lower in COVID-19-free surgical pathways in comparison to those in hospitals without defined pathways [12]. Presented in emergency circumstances, screening for COVID-19 is mandatory. Undoubtedly, a preoperative nasal swab is an important screening process prior to any surgical intervention [13]. In our case, the patient's COVID-19 screening was performed using the PCR method. However, in view of a delayed result, we proceeded with surgery with extreme caution through the use of full personal protective equipment (PPE). However, after mass vaccination, since COVID-19 has turned endemic, these circumstances have returned to the pre-COVID-19 era.

Huge abdominopelvic mass can be due to various reasons, either from surgical or gynaecological causes. Clinical symptoms and physical examination findings alone may not be adequate in helping elicit the pathology's likely origin. Ultrasound remains the first line of imaging study, especially during a pandemic, as it is readily available at the bedside, widely acceptable, inexpensive, and non-invasive [1]. The risk of viral transmission is minor among different fields should the procedure be performed at the bedside. The diagnostic accuracy is improved by combining grayscale and Doppler ultrasound with a sensitivity of 84% and a specificity of 82% in diagnosing cancer [14]. In diagnosing huge cystic lesions, even with an ultrasound, the diagnosis can be uncertain. Preoperative CT can play a role in determining the extent of the lesion, local infiltration, staging purpose, and surgical intervention [14].

In view of the tumour size, minimally invasive surgery is technically inappropriate, therefore, laparotomy was chosen as the route to retrieve the mass. Works of literature recommend an intact removal of any hydatid cyst or suspected ovarian mass to avoid any spillage of its content [15,16]. Percutaneous needle aspiration may not be a preferred option to reduce its size preoperatively due to concern of the risk of tumour seedlings if it is an ovarian malignancy and its role in hydatid cyst remains inconclusive. It was unfortunate in this case that despite a huge midline incision made, the mass could not be delivered safely in total and in fact, there was no space to allow the surgeons to confirm the origin of the mass initially. The decision for aspiration was made because an intraperitoneal rupture of the hydatid cyst would have caused a life-threatening anaphylactic reaction; hence, draining out the fluid in a controlled environment was preferred in order to facilitate mobilisation and further exploration of the abdomen. It is also essential to emphasize clear communication with an anaesthetist intraoperatively upon removing a huge abdominopelvic mass that can be associated with many life-threatening complications, predominantly after surgery owing to the rapid changes in body circulation. Since the intraoperative findings were suggestive of a benign mucinous cystadenoma with a normal-appearing uterus and contralateral ovary, the decision for a unilateral salpingo-oophorectomy was adequate. This allows for fertility preservation and it is an acceptable surgical treatment option among young women as most of the patients with similar findings are either benign or in early-stage disease [15].

Pathologic criteria are crucial in making the correct diagnosis in order not to miss the borderline or invasive components. Mucinous neoplasm represents a variety of spectrums, either benign, borderline, or invasive histologic variants. Thus, a thorough evaluation of the specimen is crucial to get a proper diagnosis. Among benign ovarian neoplasms, mucinous cystadenomas account for approximately less than 15% of all cases [17]. Mucinous cystadenomas usually occur as a large, multiloculated cystic mass with mucus-containing fluid (17). These tumours occur most commonly in women in their twenties to forties, but occurrences in adolescents, premenarchal girls, and postmenopausal patients have also been documented.

## 4 Conclusion

A giant abdominopelvic cystic mass can present emergency havoc during the COVID-19 pandemic. Ultrasound is convenient and safer, but might not be diagnostically accurate compared to a CT scan. In view of the pandemic, urgent surgical intervention is mandatory by using full PPE and exercising extreme precaution, regardless of the preoperative screening to avoid unnecessary viral transmissions.

## Ethical approval

Ethical approval was obtained from the local institution.

## Please state any sources of funding for your research

None.

## Author contribution

RG - manuscript preparation. AAA - manuscript preparation. FH - final review. VVR - literature search. AOP - data collection. AAW - literature search. NA - literature search. MMAA – data collection. BA - final review.

## Please state any conflicts of interest

The authors declare that there are no conflicts of interest.

## Registration of research studies

No ethical clearance required as it only involves case report.

## Guarantor

Nornazirah Azizan.

## Consent

Written informed consent was obtained from the patient for publication of this case report and accompanying images. Permission was also obtained from local administrators. A copy of the written consent is available on request.

## Availability of data and materials

Data sharing is not applicable to this article, as no data sets were generated or analysed during the current study.

## Provenance and peer-review

Not commissioned, externally and internally peer-reviewed.
